# Differences in Genomic Alterations and Accumulations of Heavy Metals Between Advanced Non-small Cell Lung Cancer Patients with and without Bone Metastasis

**DOI:** 10.7150/jca.95191

**Published:** 2024-06-03

**Authors:** Zhong-Qiang Yao, Hui-Hui Jiang, Fei-Fei Wang, Zhi-Gang Fan, Yi-Ge Zhang, Shang-Dong Mou, Xia Cao, Cheng-Tian Li, Li-Sha Jiang, Li Song, Shu-Shen Ji, Qing-Juan Chen

**Affiliations:** 1 Medical oncology, 3201 Hospital of Xi´an Jiaotong University Health Science Center, Hanzhong 723000, China.; 2 Zhangjiang Center for Translational Medicine, Shanghai Biotecan Pharmaceuticals Co., Ltd., Shanghai 200135, China.

**Keywords:** non-small cell lung cancer, bone metastasis, tissue, somatic mutations, somatic interactions, heavy metal

## Abstract

**Purpose:** Bone metastasis (BoM) has been closely associated with increased morbidity and poor survival outcomes in patients with non-small cell lung cancer (NSCLC). Given its significant implications, this study aimed to systematically compare the biological characteristics between advanced NSCLC patients with and without BoM.

**Methods:** In this study, the genomic alterations from the tumor tissue DNA of 42 advanced NSCLC patients without BoM and 67 patients with BoM and were analyzed by a next-generation sequencing (NGS) panel. The serum concentrations of 18 heavy metals were detected by inductively coupled plasma emission spectrometry (ICP-MS).

**Results:** A total of 157 somatic mutations across 18 mutated genes and 105 somatic mutations spanning 16 mutant genes were identified in 61 out of 67 (91.05%) patients with BoM and 37 of 42 (88.10%) patients without BoM, respectively. Among these mutated genes, *NTRK1*, *FGFR1*, *ERBB4*, *NTRK3*, and *FGFR2* stood out exclusively in patients with BoM, whereas *BRAF*, *GNAS*, and *AKT1* manifested solely in those without BoM. Moreover, both co-occurring sets of genes and mutually exclusive sets of genes in patients with BoM were different from those in patients without BoM. In addition, the serum concentrations of Cu and Sr in patients with BoM were significantly higher than in patients without BoM. One of our aims was to explore how these heavy metals associated with BoM interacted with other heavy metals, and significant positive correlations were observed between Cu and Co, between Cu and Cr, between Sr and Ba, and between Sr and Ni in patients with BoM. Given the significant impacts of molecular characteristics on patients' prognosis, we also observed a noteworthy negative correlation between *EGFR* mutations and Co, alongside a significant positive correlation between *TP53* mutations and Cd.

**Conclusions:** The genomic alterations, somatic interactions, key signaling pathways, functional biological information, and accumulations of serum heavy metals were markedly different between advanced NSCLC patients with and without BoM, and certain heavy metals (e.g., Cu, Sr) might have potentials to identify high-risk patients with BoM.

## Introduction

As the foremost contributor to cancer-related deaths globally, lung cancer accounted for approximately 18% of these deaths 1. Non-small-cell lung cancer (NSCLC) constitutes nearly 80% of lung cancer cases, comprising subtypes such as adenocarcinoma (LUAD), squamous carcinoma (LUSC), adenosquamous carcinoma, large cell carcinoma, and sarcomatoid carcinoma 2. An estimated 30-40% of NSCLC patients develop bone metastasis (BoM) at initial diagnosis 3, and suffer from hypercalcemia, pathological fractures, spinal instability, spinal compression, bone pain, and other skeletal-related events (SREs). These SREs compromise bone structural integrity and often serve as ominous indicators 4, 5. Following the onset of BoM, patients with NSCLC typically face a median survival period of 6-8 months 6.

Given the significant impact of BoM on patients' prognosis, it is imperative to identify risk factors for the occurrence of BoM in patients with lung cancer. This not only aids in expeditious and accurate diagnosis but also facilitates the anticipation of its progression, predating existing imaging methods (e.g., skeletal scintigraphy, computerized tomography (CT), Positron emission tomography-computed tomography (PET-CT), magnetic resonance imaging (MRI)) 7-9. So far, the incidence of BoM has shown positive correlations with various factors such as male gender, married status, younger age (≤50), adenocarcinoma type, clinical stage (III-IV), TNM stage (T1-T3, N2-N3), fibrinogen, activated partial thromboplastin time, D-Dimer, alkaline phosphatase, metabolic tumor volume (MTV) of the whole body (MTVwb), and MTV of thorax (MTVtho) 3, 10-15. Intriguingly, patients with primary lung tumors in the lower lobe exhibited a higher propensity for BoM compared to those with tumors in the main bronchus, suggesting that different primary tumor locations may influence the pattern of distant metastasis in patients with advanced NSCLC 16. For the bone-matrix signaling, NSCLC patients with BoM had significantly elevated serum levels of bone sialoprotein (BSP) compared to patients without BoM or control subjects 17. Moreover, a strong correlation was observed between BSP expression and BoM in the primary resected NSCLC patients 18.

Disruptions in metal homeostasis can trigger the activation of oncogenic signaling pathways, hinder the DNA repair system, induce oxidative stress, and modify epigenetic inheritance 19-22. Individuals with lung cancer exhibited higher concentrations of copper (Cu) and elevated Cu/Zn ratios in serum compared to healthy controls 23-25. Lung cancer patients often displayed increased levels of ferritin in their serum and bilateral bronchoalveolar lavage fluid 26. Moreover, the expression of serum ferritin (SF) showed a significant positive correlation with regional lymph node metastasis and distant metastasis 27. For patients with small cell lung cancer, zinc finger E-box binding homeobox 1 was found to be highly expressed in bone-metastatic tissues compared to those in non-metastatic tissues 28. In addition, advanced NSCLC individuals with hyponatremia tended to develop BoM significantly earlier than those without hyponatremia 29. Lower blood calcium levels have also been associated with an unfavorable prognosis and the potential to predict the occurrence of BoM in patients with NSCLC 30. However, there is currently insufficient evidence to suggest that these above factors possess a robust predictive capability for BoM. Consequently, it is meaningful to systematically compare the biological characteristics between advanced NSCLC patients with and without BoM.

In this study, targeted next-generation sequencing (NGS) of 95 tumor-associated genes and inductively-coupled plasma mass spectrometry (ICP-MS) detection of 18 heavy metals were performed on tissue DNA and serum samples from patients with advanced NSCLC, respectively. This study aimed to explore: (i) the differences in genomic alterations, somatic interactions, and KEGG and GO signaling pathways between advanced NSCLC patients with and without BoM, (ii) which of serum heavy metals is correlated with BoM in patients with advanced NSCLC, (iii) how do these heavy metals associated with BoM interact with somatic mutations, demographic and clinical characteristics, and other heavy metals in patients with BoM.

## Materials and methods

### Patients and sample collection

A total of 109 patients with advanced NSCLC from the Medical oncology at 3201 Hospital of Xi´an Jiaotong University Health Science Center were recruited between November 2020 and January 2023. The pathological diagnosis was verified by three pulmonary pathologists, following the criteria laid out in the 4th edition of the World Health Organization Classification of Lung Tumors 31. In particular, the diagnosis of bone metastasis adhered to the guidelines set by the National Comprehensive Cancer Network (NCCN), which include clinical signs and symptoms (e.g., ostealgia, progressive limitation of movement or mobility, local tenderness or tenderness under physical contact or oppression), imageological examinations (e.g., bone scintigraphy, computed tomography, magnetic resonance imaging), or/and the biopsy of bone metastatic lesion 7, 8. A total of 109 formalin-fixed and paraffin-embedded (FFPE) tumor specimens were collected for the high-throughput analysis. Among this cohort of 109 patients, 91 individuals underwent heavy metal detection using serum samples. Written informed consent was obtained from all participants, and this study was conducted in strict accordance with the Code of Ethics of the World Medical Association (Declaration of Helsinki) 32. Furthermore, this study received approval from the Medical Ethics Committee of 3201 Hospital of Xi´an Jiaotong University Health Science Center, and its Institutional Review Board (IRB) number was No.017(2020).

Inclusion criteria: (1) Age > 18 years old; (2) First diagnosis of NSCLC by histology examination; (3) The availability of enhanced CT scan of the chest and abdomen, magnetic resonance imaging (MRI) of the brain, and whole-body bone scan (ECT) results; (4) Primary NSCLC with distant metastasis; (5) Without any treatments.

Exclusion criteria: (1) NSCLC tumors displaying histological components other than LUAD and LUSC; (2) Recurrence; (3) Contraindication to an enhanced CT scan, a cranial MRI, or ECT examination; (4) Exposure history of trace elements, toxic elements, or heavy metals; (5) Patients consumed antioxidants, vitamins, or dietary supplements.; (6) Patients had suffered from the surgery within the past year; (7) Patients had comorbidities such as autoimmune disease, diabetes mellitus, gout, hypoglycemia, hypertension, heart disease, chronic liver disease, chronic kidney disease, protein-energy malnutrition, thyroid disease, and vitamin A/D deficiency; (8) Patients had additional conditions deemed inappropriate for this study by our research team.

### DNA extraction and quality control

Genomic DNA (gDNA) was extracted from FFPE tumor specimens employing the GeneRead DNA FFPE kit from Qiagen GmbH. Subsequently, the quantity and purity of the gDNA were assessed using Qubit® 3.0 Fluorometer (Invitrogen; Thermo Fisher Scientific, Inc.) and NanoDrop ND-1000 (Thermo Scientific, Inc.), respectively. To evaluate its integrity, quality control (QC) procedures were conducted using a multiplex Polymerase Chain Reaction (PCR) approach.

### Library preparation, hybridization capture, and Illumina sequencing

Three hundred nanogram (ng) of gDNA underwent mechanical fragmentation via an E220-focused ultrasonicator Covaris (Covaris, LLC.). The target fragment size ranged from 150 to 200 base pairs (bp). Subsequently, DNA samples ranging from 10 to 100 ng were employed for library construction, following the manufacturer's guidelines using the KAPA library preparation kit (Kapa Biosystems Inc.; Roche Diagnostics). This process encompassed end-repair, A-tailing, and adapter ligation without the need for additional fragmentation. The NGS libraries were subjected to capture using the xGen Lockdown Probe pool sourced from Integrated DNA Technologies, Inc. The captured DNA fragments were amplified through 13 cycles of PCR, utilizing 1X KAPA HiFi Hot Start Ready Mix (Kapa Biosystems Inc.; Roche Diagnostics). Then, quality control and quantification were conducted, employing the Agilent 2100 Bioanalyzer (Agilent Technologies, Inc.) and the Qubit® 3.0 Fluorometer (Invitrogen; Thermo Fisher Scientific, Inc.). Finally, the NGS libraries were sequenced utilizing an Illumina NextSeq CN500 platform equipped with a medium flux chip (NextSeq CN500 Mid Output v2 kit; Illumina Inc.).

### Bioinformatics analysis

Clean data were obtained by filtering out low-quality reads, which encompassed reads containing adapter sequences and those with a length of less than 36 bp. All of the filtered reads underwent alignment to the human genome (University of California Santa Cruz ID: hg19), employing the Burrows-Wheeler-Alignment Tool (BWA v.0.7.12; Wellcome Trust Sanger Institute) 33. Then, we implemented the Picard and Genome Analysis Toolkit (GATK v.3.2) methodology for a series of essential processes, including the removal of duplicate sequences, local realignment, and recalibration of base quality scores. This comprehensive approach was also utilized for the generation of quality statistics. Finally, the VarDict tool (v.1.6.0) (GitHub, Inc.) was adopted for the systematic identification of single nucleotide variations (SNVs) and Insertions/Deletions (InDels) 34.

The ANNOVAR software tool (v. 20210202; https://annovar.openbioinformatics.org/en/latest/) was employed for the annotation of somatic variants 35. The identification of candidate somatic variants adopted the following filter conditions: i) Variants with a coverage depth (VDP) of less than 10 were excluded; ii) Variant sites with a mutant allele frequency (MAF) greater than 0.001 in the 1,000 Genomes databases (1,000 Genomes Project Consortium; https://www.internationalgenome.org/) and East Asian in Exome Aggregation Consortium (ExAC_EAS) (https://gnomad.broadinstitute.org/) were removed; iii) Variant sites with MAF between 0.001 and 0.1 in the 1,000 Genomes databases that had COSMIC evidence (http://cancer.sanger.ac.uk/cosmic) were retained; iv) Variations located in the exon or splicing region (10 bp upstream and downstream of splicing sites) were retained; v) Synonymous mutations were removed; vi) Variants with unknown classifications were excluded; vii) Functional benign variant sites predicted by Polymorphism Phenotyping v2 (PolyPhen 2; http://genetics.bwh.harvard.edu/pph2/) were removed 36; viii) Polymorphism and automatic polymorphism variant sites predicted by MutationTaster (MutationTaster2020; https://www.mutationtaster.org/) were removed 37; ix) Neutral and unknown variant sites predicted by LRT (dbNSFP version 3.0; http://sites.google.com/site/jpopgen/dbNSFP) were excluded.

### ICP-MS Detection

Serum samples were collected from 91 patients and subjected to heavy metal detection by ICP-MS (Agilent 7800). The serum concentrations of 18 heavy metals were evaluated, Arsenic (As), Barium (Ba), Cadmium (Cd), Cobalt (Co), Chromium (Cr), Cuprum (Cu), Gallium (Ga), Mercury (Hg), Manganese (Mn), Nickel (Ni), Plumbum (Pb), Stibium (Sb), Selenium (Se), Stannum (Sn), Strontium (Sr), Thallium (Tl), Vanadium (V), and Zinc (Zn). The precise detection protocol of ICP-MS adhered to the manufacturer's instructions (35). Briefly, a minimum of 2 milliliters of whole blood from each patient underwent centrifugation at 3000 revolutions per minute for 10 minutes to obtain the upper serum, which was stored at -20°C.

### Statistical analysis

The analysis of somatic mutation landscapes, co-barplots, co-oncoplots, lollipop plots, co-occurring and mutually exclusive genomic alterations, Kyoto Encyclopedia of Genes and Genomes (KEGG) enrichment, and Gene Ontology (GO) enrichment was performed by R software (version 4.0.3, R Core Team; https://www.R-project.org) 38. To assess statistical differences, Fisher's exact test and the Mann-Whitney test were employed for categorical and continuous variables, respectively. A significance level of P<0.05 was used to determine statistically significant differences. Additionally, spearman correlation analysis was applied to investigate the associations among BoM, *EGFR* mutations, *TP53* mutations, 9 demographic and clinical characteristics, and 18 heavy metals.

## Results

### Patient characteristics

In this retrospective study, 109 patients with advanced NSCLC were recruited including 67 patients with BoM (aged 47-83 years) and 42 patients without BoM (aged 45-83 years). No significant differences in the demographic and clinical characteristics were found between patients with and without BoM except for the expression of CA-199 (P=0.018) and CEA (P=0.035) (Table 1).

### Comparison of somatic mutations between advanced NSCLC patients with and without BoM

**Figure 1:**
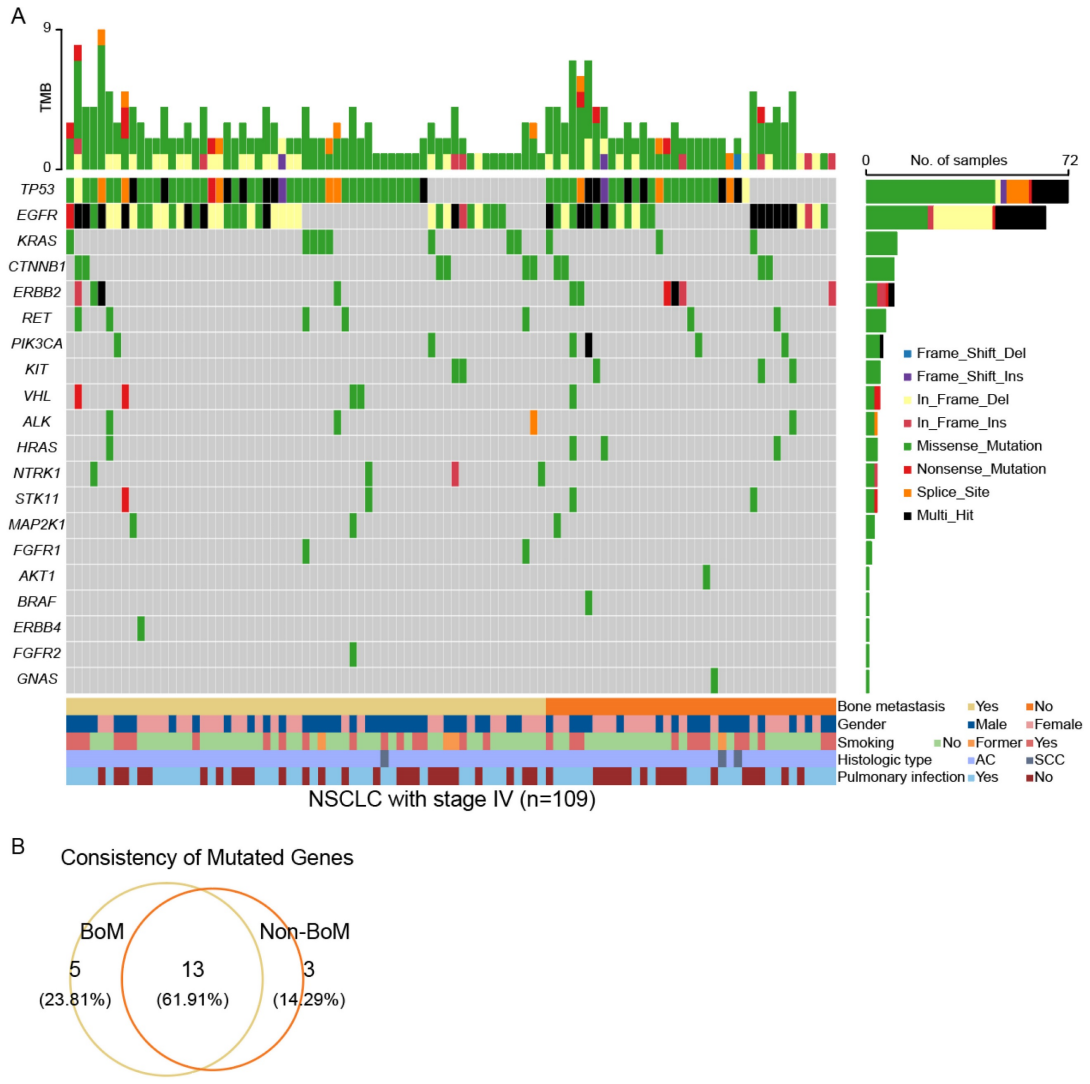
The landscapes of somatic mutations for advanced NSCLC patients with BoM (named BoM group, n=67) and without BoM (named Non-BoM group, n=42) **(A)**. Venn diagram of mutant genes derived from these two groups **(B)**.

To elucidate the disparities in genomic alterations between patients with and without BoM, we conducted an analysis of somatic mutations in a panel of 95 cancer-related genes (Table S1). A total of 157 somatic mutations across 18 mutant genes in 61 out of 67 (91.05%) patients with BoM (named BoM group) were identified. In contrast, 105 somatic mutations involving 16 mutant genes were detected in 37 out of 42 (88.10%) patients without BoM (named Non-BoM group) (Fig. 1A and Table S2). To delve further into the molecular distinctions between these two groups, we generated a Venn diagram (Fig. 1B). Among these 21 mutated genes, *NTRK1*, *FGFR1*, *ERBB4*, *NTRK3*, and *FGFR2* were exclusively identified in patients with BoM, whereas *BRAF*, *GNAS*, and *AKT1* were unique to patients without BoM. In addition, *EGFR*, *KRAS*, and 11 other mutant genes concurrently presented in both groups.

### Comparison of somatic interactions between advanced NSCLC patients with and without BoM

**Figure 2:**
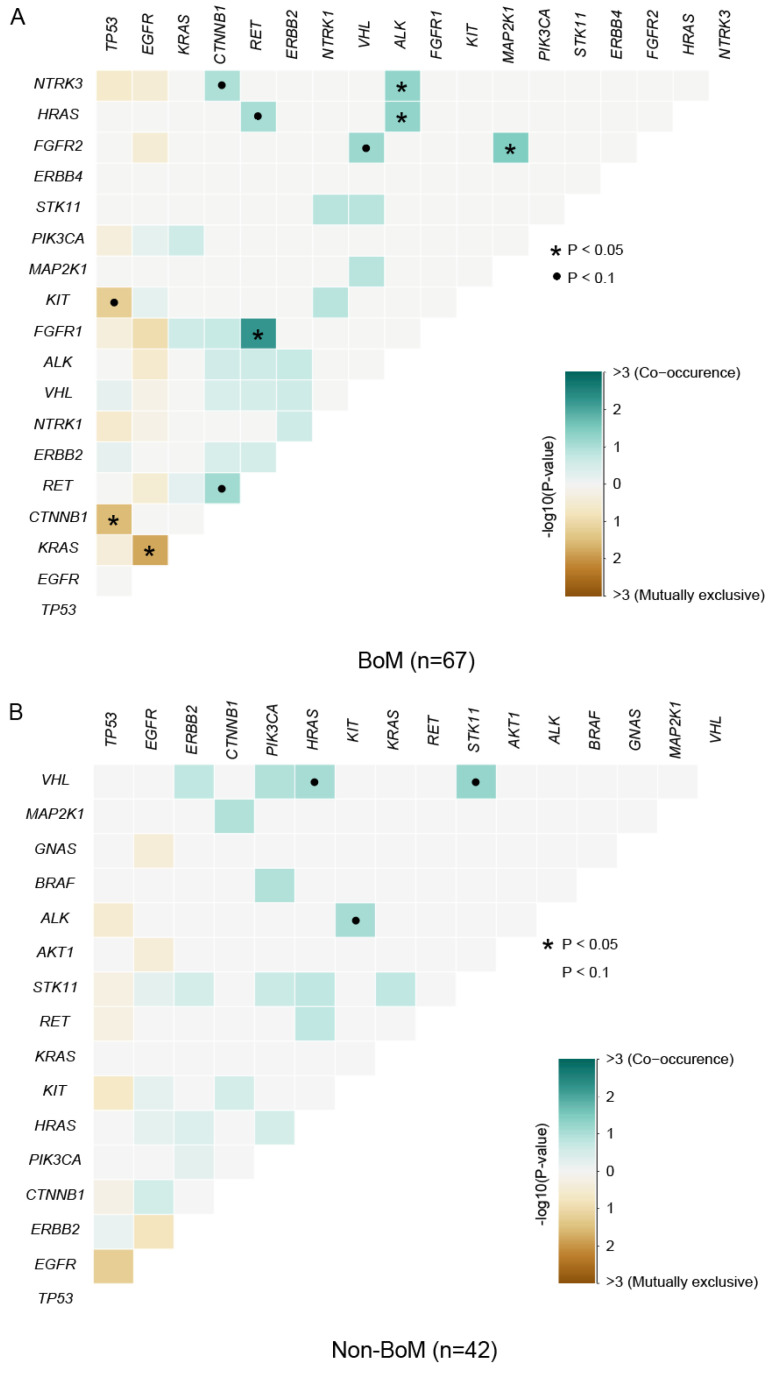
The Spectrum of co-occurring and mutually exclusive mutant genes in the BoM group (n=67) **(A)** and the non-BoM group (n=42) **(B)**.

In LUAD, *EGFR* and *KRAS* mutations are typically found to be mutually exclusive, and the presence of *KRAS* mutations can confer resistance to EGFR-Tyrosine Kinase Inhibitors (TKIs), such as gefitinib and erlotinib 39. In this study, the pattern of somatic interactions markedly differed between patients with and without BoM. Co-occurring interactions were identified between *FGFR1* and *RET* (P=0.0053), between *FGFR2* and *MAP2K1* (P=0.0323), and six other sets of genes in patients with BoM (P<0.1) (Fig. 2A and Table S3), whereas between *STK11* and *VHL* (P=0.0526), between *VHL* and *HRAS* (P=0.0789), between *ALK* and *KIT* (P=0.0789) were three co-occurring sets of genes in patients without BoM (Fig. 2B and Table S3). Meanwhile, marked distinctions were also observed in the mutually exclusive sets of genes between these two groups. As depicted in Fig. 2, mutual exclusivity was identified between *KRAS* and *EGFR* (P=0.0193), between *CTNNB1* and *TP53* (P=0.0340), and between *TP53* and *KIT* (P=0.0635) in patients with BoM, while no mutually exclusive interactions were found in patients without BoM (P≥0.1).

### Comparison of Key signaling pathways and biological functions between advanced NSCLC patients with and without BoM

To gain a more comprehensive understanding of the biological implications in both groups, we conducted KEGG and GO enrichment analyses. As shown in supplementary figures 1A and 1B, a notable emphasis on cancer-related signaling pathways was observed (Table S4). Interestingly, all KEGG pathways enriched in patients with BoM were observed in patients without BoM (Figure S1A, S1B, and S1E, top). Moreover, toll-like receptor signaling pathways, carbohydrate digestion and absorption, and other five KEGG pathways were exclusively in patients without BoM. In GO enrichment analysis, the functional categories were prominently associated with transmembrane receptor protein tyrosine kinase activity in patients with BoM (Figure S1C and Table S4) and protein tyrosine kinase activity in patients without BoM (Figure S1D and Table S4). Specifically, ephrin receptor activity, fibroblast growth factor binding, SH2 domain binding, heparin binding, and neurotrophin receptor binding were uniquely enriched in patients with BoM (Figure S1E, down).

### Comparison of Clinical actionability of targeted drug therapy between advanced NSCLC patients with and without BoM

**Figure 3:**
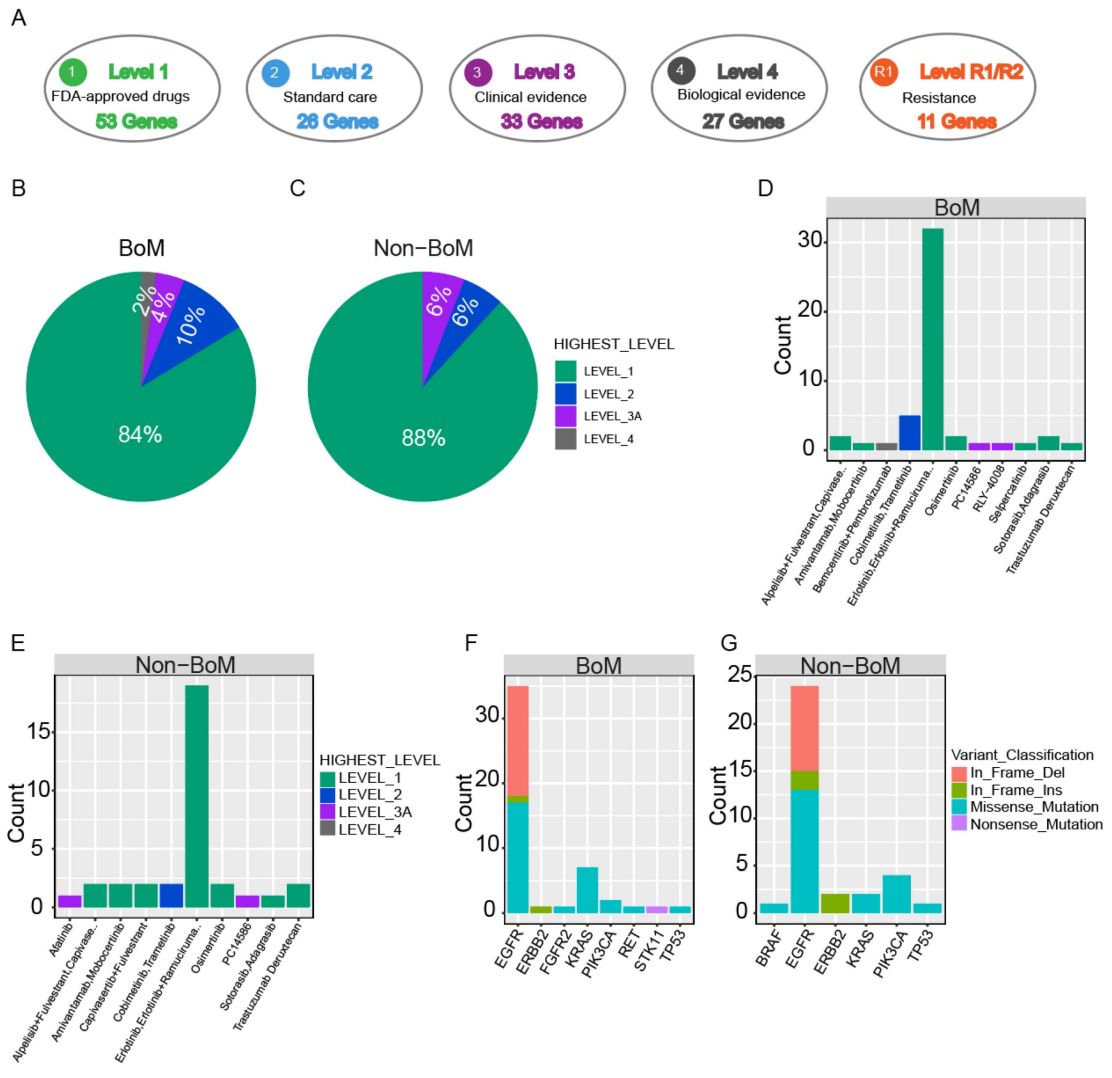
Clinical actionability of somatic mutations revealed by a hybridization capture-based NGS panel. Somatic mutations were characterized according to their clinical evidence as outlined by OncoKB (A). Samples were matched to the highest level of actionable alterations for the BoM group **(B)** and the non-BoM group **(C)**. Distribution of actionable alterations **(D, E)** and mutation type **(F, G)** in the BoM group **(D, F)** and the non-BoM group **(E, G)**.

To evaluate the practicality of anticipatory molecular profiling, mutations were categorized into distinct tiers based on their clinical relevance as indicated in OncoKB (Fig. 3A). As standard therapeutic indicators, a group of gene mutations had been approved by the FDA. In patients with BoM, 67.16% (45/67) of patients possessed at least one actionable alteration. Among these actionable alterations, level_1 accounted for 84.00%, including Missense Mutation of *EGFR*, *PIK3CA*, *KRAS*, and *RET*, In-frame Insertion of *EGFR* and *ERBB2*, and In-frame Deletion of *EGFR*; level_2 accounted for 10.00%, including Missense Mutation of *KRAS*; level_3 accounted for 4.00%, including Missense Mutation of *TP53* and *FGFR2*; level_4 accounted for 2.00%, including Nonsense Mutation of *STK11* (Fig. 3B, D, and F, and Table S5). For patients without BoM, 66.67% (28/42) of patients exhibited at least one actionable alteration. Among these actionable alterations, level_1 constituted 88.00%, encompassing Missense Mutations in *EGFR*, *PIK3CA*, and *KRAS*, In-frame Insertions in *EGFR* and *ERBB2*, and In-frame Deletions in *EGFR*; level_2 constituted 6.00%, encompassing Missense Mutations in *BRAF* and *KRAS*; level_3 constituted 6.00%, encompassing Missense Mutation in *TP53*, and In-frame Deletion in *EGFR* (Fig. 3C, E, and G). Collectively, similar percentages of actionable alterations were observed between patients with and without BoM, suggesting both groups derived significant benefits from targeted therapies.

### Comparison of Accumulations of Serum Heavy Metals between advanced NSCLC patients with and without BoM

**Figure 4:**
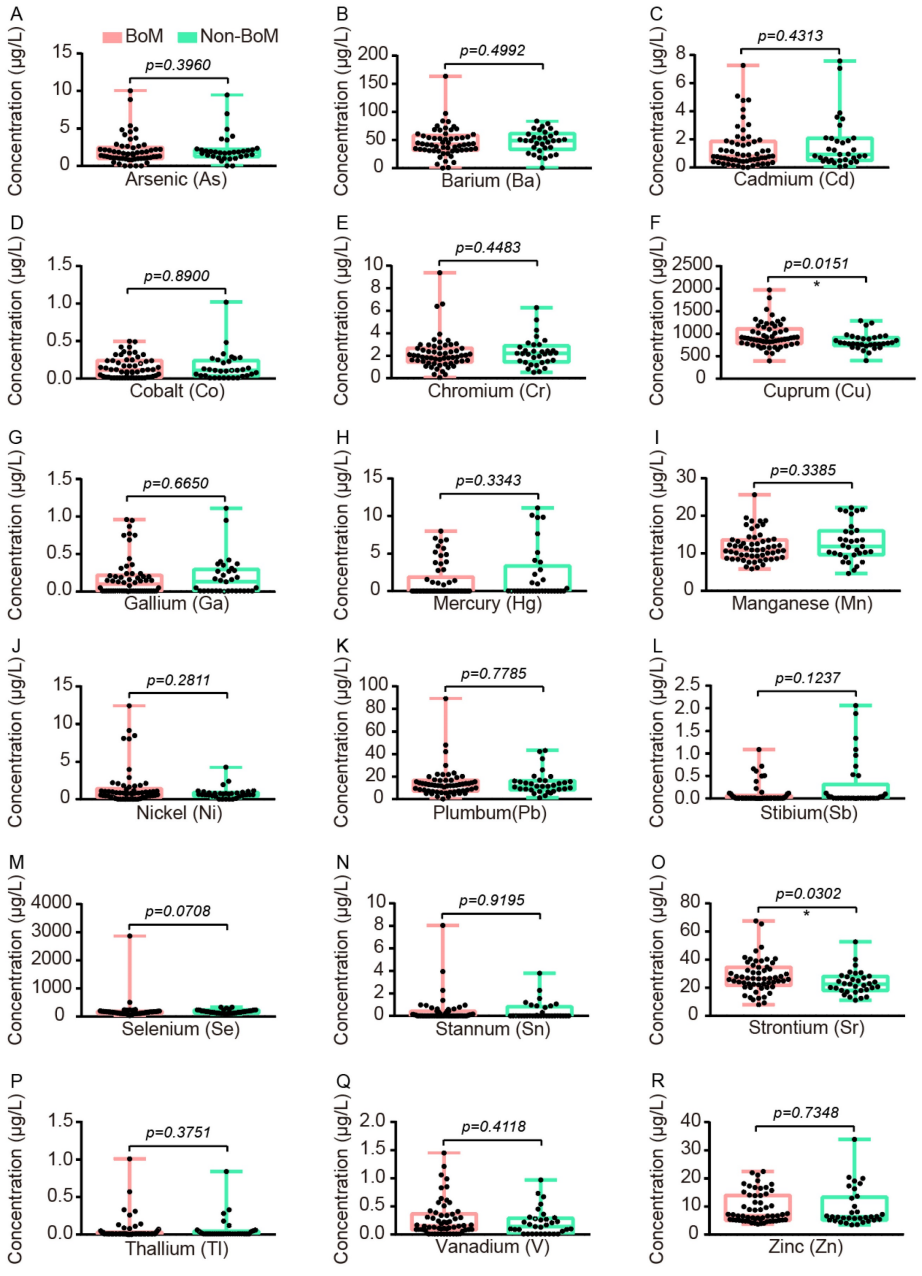
The comparative analysis of 18 heavy metals between the BoM group (n=58) and the non-BoM group (n=33), including As **(A)**, Ba **(B)**, Cd **(C),** Co **(D)**, Cr **(E)**, Cu **(F)**, Ga **(G)**, Hg **(H)**, Mn **(I)**, Ni **(J)**, Pb **(K)**, Sb **(L)**, Se **(M)**, Sn **(N)**, Sr **(O)**, Tl **(P)**, V **(Q)**, and Zn **(R)**. Statistical analysis was performed by the Two-tailed Mann Whitney U test. * p < 0.05.

To explore which serum heavy metals were associated with BoM in patients with advanced NSCLC, we conducted heavy metal detection in the serum samples of 58 patients with BoM and 33 patients without BoM. The levels of Cu and Sr in patients with BoM were significantly higher than those in patients without BoM, and their median concentrations with interquartile ranges (IQR) were as follows: Cu 893.3 (783.9-1108) vs. 791.2 (739.8-913.9) μg/L (P=0.0151), and Sr 26.52 (21.74-34.55) vs. 22.51 (18.10-28.00) μg/L (P=0.0302) (Fig. 4).

### Correlation Analysis Among BoM, Genomic Alterations, Demographic and Clinical Characteristics, and Heavy metals in patients with advanced NSCLC

**Figure 5:**
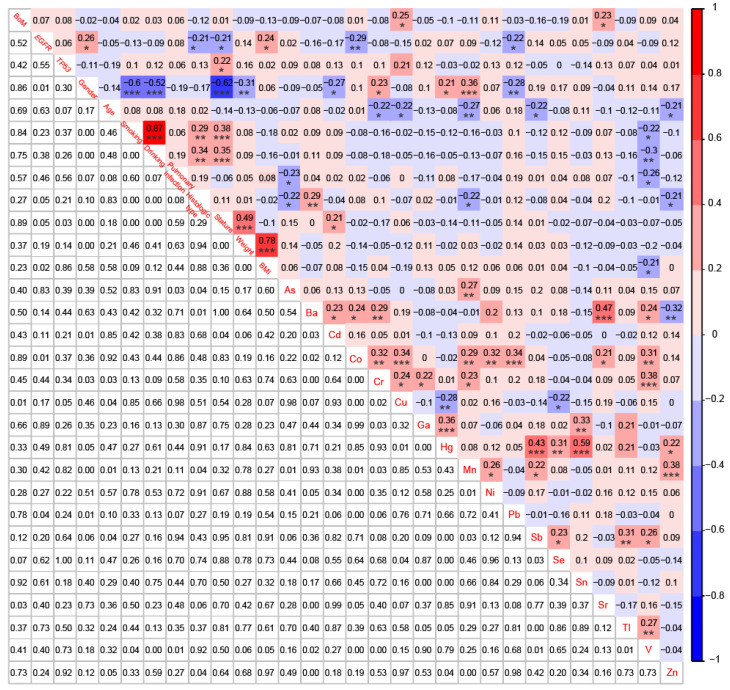
Correlations among BoM, *EGFR* mutations, *TP53* mutations, 9 demographic and clinical characteristics, and 18 heavy metals in patients with advanced NSCLC (n=91). * p < 0.05, ** p < 0.01, and *** p < 0.001.

To establish correlations among BoM, *EGFR* mutations, *TP53* mutations, 9 demographic and clinical characteristics, and 18 heavy metals, we simultaneously conducted NGS analysis and heavy metal detection on 58 patients with BoM and 33 patients without BoM. BoM was significantly positively correlated with the concentrations of Cu (r = 0.25, *P* < 0.05) and Sr (r = 0.23, *P* < 0.05), whereas no significant correlations were observed between BoM and genomic alterations, demographic characteristics, or clinical characteristics (Fig. 5). Female was significantly negatively correlated with the history of smoking and drinking, stature, and weight, which was consistent with previous studies 40. Meanwhile, female displayed negative correlations with Cd (r = -0.27, *P* < 0.05) and Pb (r = -0.28, *P* < 0.01), whereas significantly positive correlations were observed between female and Cr (r = 0.23, *P* < 0.05), Hg (r = 0.21, *P* < 0.05), or Mn (r = 0.36, *P* < 0.001). What's more, age displayed significantly negative correlations with Cr (r = -0.22, *P* < 0.05), Cu (r = -0.22, *P* < 0.05), Mn (r = -0.27, *P* < 0.01), Sb (r = -0.22, *P* < 0.05), and Zn (r = -0.21, *P* < 0.05). Lastly, significant correlations were also identified between most of the 18 heavy metals, such as between Ba and Sr (r = 0.47, *P* < 0.001), between Hg and Sb (r = 0.43, *P* < 0.001), between Hg and Sn (r = 0.59, *P* < 0.001), and others (Fig. 5).

### Correlation Analysis Among Genomic Alterations, Demographic and Clinical Characteristics, and Heavy metals in NSCLC patients with BoM

**Figure 6:**
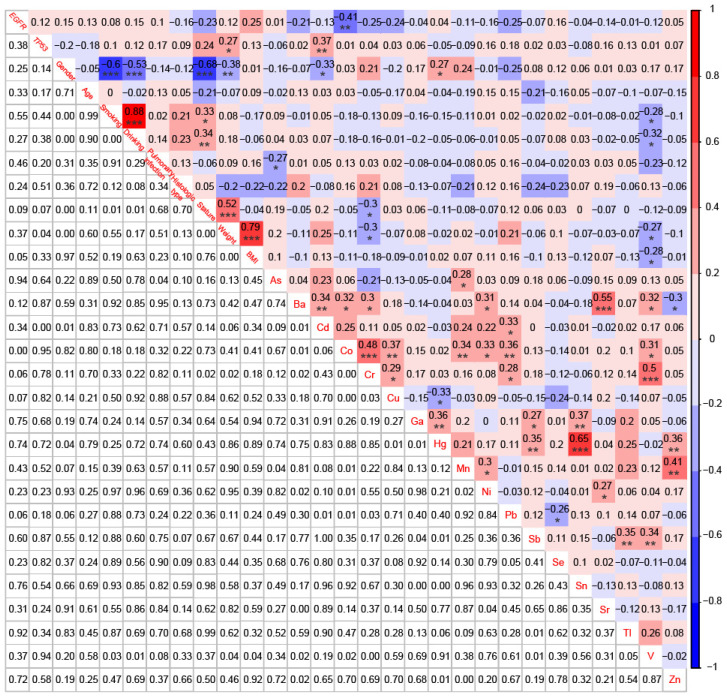
Correlations among *EGFR* mutations, *TP53* mutations, 9 demographic and clinical characteristics, and 18 heavy metals in patients with BoM (n=58). *p < 0.05, **p < 0.01, and ***p < 0.001.

For 58 patients with BoM, except for the significant negative correlation between Cu and Hg (r = -0.33, *P* < 0.05), significant positive correlations were identified between Cu and Co (r = 0.37, *P* < 0.01), between Cu and Cr (r = 0.29, *P* < 0.05), between Sr and Ba (r = 0.55, *P* < 0.001), and between Sr and Ni (r = 0.27, *P* < 0.05) (Fig. 6). However, neither Cu nor Sr did show any significant correlation with *EGFR* mutations, *TP53* mutations, or the 9 Demographic and Clinical Characteristics. Meanwhile, the serum concentration of Co was significantly negatively correlated with *EGFR* mutations (r = -0.41, *P* < 0.01), whereas Cd displayed a significant positive correlation with* TP53* mutations (r = 0.37, *P* < 0.01).

In addition, for 33 patients without BoM, *EGFR* mutations were significantly negatively correlated with the history of smoking (r = -0.50, *P* < 0.01) and the history of drinking (r = -0.53, *P* < 0.01), and notably positively correlated with Mn (r = 0.43, *P* < 0.05) and Sb (r = 0.50, *P* < 0.01) (Figure S2). No significant correlations were found between *TP53* mutations and the 9 demographic and clinical characteristics, but *TP53* mutations were significantly positively correlated with As (r = 0.43, *P* < 0.05) and Cu (r = 0.50, *P* < 0.01) (Figure S2).

## Discussion

While dynamic differences in clinical characteristics have been reported between patients with and without BoM, systematic comparisons of the genomic alterations, somatic interactions, and accumulations of serum heavy metals have been lacking. In this study, we identified 157 somatic mutations across 18 mutated genes in 61 out of 67 (91.05%) patients with BoM and 105 somatic mutations in 16 mutant genes in 37 out of 42 (88.10%) patients without BoM by an NGS panel. Significant disparities in genomic alterations, somatic interactions, key signaling pathways, and functional biological insights were found between these two groups. Moreover, the detection of 18 heavy metals was performed on serum samples from 58 patients with BoM and 33 patients without BoM, and the concentrations of Cu and Sr were significantly higher in patients with BoM compared to those without. Notably, BoM exhibited significant positive correlations with Cu and Sr in patients with advanced NSCLC, whereas no significant correlations were observed between BoM and genomic alterations, demographic characteristics, or clinical characteristics. Finally, significant positive correlations were identified between Cu and Co, between Cu and Cr, between Sr and Ba, and between Sr and Ni in patients with BoM.

While the impact of tumor mutation status on BoM in NSCLC has been studied, there is no consensus yet 41. In a retrospective case-control study involving 189 metastatic NSCLC patients, no significant differences were observed in the incidence of BoM, mean time to develop BoM, or time to first SRE among patients with *EGFR* (exon 19 and 21) mutations, *KRAS* mutations, and wild type *EGFR*/*KRAS* 42. Similarly, in a study involving 209 NSCLC patients with *EGFR*, *KRAS*, *ALK*, or without mutations at diagnosis, no specific molecular group showed a predisposition to the development of BoM 43. Consistent with previous studies, both *EGFR* (59.70% vs. 57.14%) and *KRAS* (11.94% vs. 7.14%) mutation frequencies were similar between patients with and without BoM in our study. However, a retrospective study involving 570 patients with NSCLC reported that patients harboring *EGFR* and *HER2* alterations showed a heightened incidence of lung and bone metastases compared to those with gene fusions, *RAS*/*RAF* mutations, or mutations lacking a known driver oncogene 44. In a retrospective study involving 246 patients with advanced LUAD, patients harboring *EGFR* mutations exhibited a significantly greater number of metastatic lesions in the bone compared to wild-type patients 45. Moreover, the median overall survival (OS) was extended in patients with *EGFR* mutations than in wild-type patients, possibly attributed to the availability of effective targeted therapies for *EGFR*-mutated NSCLC 42, 45. Collectively, contradictory findings exist, and future studies should not only expand the sample size but also focus on more mutant genes, such as exclusive mutations related to BoM.

Among these mutated genes in this study, *NTRK1*, *FGFR1*, *ERBB4*, *NTRK3*, and *FGFR2* were exclusively present in patients with BoM, while *BRAF*, *GNAS*, and *AKT1* were specific to patients without BoM. Aberrant fibroblast growth factor receptor (*FGFR*) signaling is a common feature in various cancer types 46-51. Amplifications are the most prevalent *FGFR1-4* genomic alterations, with missense mutations in FGFR being relatively rare 52, 53. For NSCLC, previous studies have reported a 6% amplification rate of *FGFR1* 54, which contrasts with our findings. The differences in mutation types of *FGFR1* could be attributed to variations in patients' demographic and clinical characteristics, such as ethnicity, region, anatomical stage, and distant metastasis. FGFR plays a crucial role in direct interactions with cell adhesion molecules (CAMs) and extracellular matrix (ECM) proteins, contributing to the invasive and migratory properties of cancer cells, whereas interactions with other receptor tyrosine kinases (RTKs) regulate angiogenesis, resistance to therapy, and metastatic potential of cancer cells 55-61. Although the direct interactors of FGFR1/2 were unrecognized in this study, three co-occurring sets of genes in patients with BoM were identified, including between *FGFR1* and *RET*, between *FGFR2* and *MAP2K1*, and between *FGFR2* and *VHL*. Interestingly, FGFR-RAS-MAPK signaling has been studied well in multiple cancers 62-66, but there was a lack of validation in NSCLC patients with BoM. Previous research by Tirtha and Ross has demonstrated the interaction of the RET domain of the RET-kinesin family member 5B (RETKIF5B) fusion protein with FGFR and EGFR in endocytic RAB vesicles, contributing to invadopodia formation 67. Inhibition of FGFR or EGFR, combined with the RET inhibitor sorafenib, significantly improved the response to treatment in human cancer cell lines harboring the RET-HIF5B fusion protein 67, indicating the therapeutic potential associated with these co-occurring interactors. Collectively, our findings may not only contribute to a more comprehensive understanding of pathogenesis for NSCLC patients with BoM but also provide prospective therapeutic targets for this specific subgroup.

Various studies have highlighted the strong link between NSCLC and heavy metals. Mu *et al.* have reported that patients with LUSC showed a significant positive correlation with the serum concentration of Ba, whereas this correlation was not observed in LUAD 40. However, limited research has delved into the impact of heavy metals on the progression of BoM. In NSCLC, lower blood calcium levels indicated an unfavorable prognosis and had the potential to predict BoM 30. Additionally, hyponatremia also emerged as a negative prognostic factor in NSCLC, and patients with hyponatremia developed BoM significantly earlier than those without hyponatremia 29. Unfortunately, our study focused on exploring the biological effects of certain heavy metals, As, Ba, Cd, Co, Cr, Cu, Ga, Hg, Mn, Ni, Pb, Sb, Se, Sn, Sr, Tl, V, and Zn---excluding calcium and sodium due to detection limitations. In this study, the concentrations of serum Cu and Sr in patients with BoM were significantly higher than those in patients without BoM. Cu, a crucial mineral nutrient, possesses a dual nature—both beneficial and potentially toxic to cells due to its inherent oxidation-reduction (redox) properties. Noteworthy, the silencing of the copper transporter ATP7A could attenuate Lysyl oxidase (LOX) activity and metastasis of Lewis lung carcinoma cells in mice 68. Cu was also essential for the activity of autophagic kinases ULK1 and ULK2 (ULK1/2), promoting tumor growth and progression in a mouse model of Kras^G12D^-driven LUAD 69. However, it remains unknown whether this autophagic regulation of the Cu-ULK1/2 interaction is implicated in the progression of BoM.

Furthermore, the interplay among various heavy metals also plays pivotal roles in tumorigenesis and progression. In patients with lung cancer, significant positive correlations were observed between the serum levels of Cd and Pb, between Mn and Fe, and between As and Pb, whereas a significant negative correlation was identified between Co and Mg, and between Ba and Sb 40, 70. Delving into the antineoplastic effects of As, its primary reliance on inducing apoptosis in squamous cell carcinomas through the generation of reactive oxygen species (ROS) and activation of JNK1/2 and caspase-3 was found 71, 72. Intriguingly, the inhibitory impact of Ba on As-induced apoptosis was noted, potentially promoting tumor progression in individuals exposed to both As and Ba 73. One of our aims was to explore how these heavy metals (Cu, Sr) associated with BoM interacted with other heavy metals, and significant positive correlations were observed between Cu and Co, between Cu and Cr, between Sr and Ba, and between Sr and Ni in patients with BoM. However, although Cu was strongly positively correlated with Cr in our study, this correlation was not found in the art glass industry workers with lung cancer 74, partly due to the differences in sample collection, occupation, and region. Given the significant impact of molecular characteristics on patients' prognosis, we also observed a noteworthy negative correlation between *EGFR* mutations and Co, as well as a significant positive correlation between *TP53* mutations and Cd. However, our findings, as of now unreported, warranted further exploration to unravel their implications and significance in the broader context of NSCL research.

Lastly, this study had its limitations, primarily manifesting in four aspects. Firstly, NGS data were exclusively obtained from primary tumor tissue samples, lacking corresponding normal tissue samples. This choice was guided by the sufficiency of actionable alterations for clinical decision-making by a single sample under appropriate filter conditions 37, 75-82. Meanwhile, it was crucial to acknowledge that the expense associated with obtaining multi-type or multiregional biopsies far surpassed that of a singular sample. Secondly, mutational differences between primary tumor lesions and matched metastatic lesions were lacking. One reason is the high expense of multiregional biopsies, the other is the generally high consistency of mutation patterns between primary lung cancer lesions and matched bone metastases 83. Thirdly, our examination focused solely on the total concentrations of each heavy metal in serum, lacking a detailed exploration of the varied forms of individual metals and their sources of exposure (e.g., soil, food, water, or air). Fourthly, the concentrations of heavy metals were exclusively assessed in serum, neglecting paired urine, hair, and nail samples. Hair and nails serve as direct repositories for heavy metals (e.g., Co, Cr, Cu, Fe, Ni, and Zn), rendering them suitable for monitoring the impact of heavy metals on health. Future studies would benefit from concurrently analyzing the concentrations of heavy metals in serum, hair, and nail samples from one individual. Lastly, to enhance the robustness of our conclusions, it is imperative to conduct further studies with larger sample sizes and multi-institutions for validation and generalizability.

## Conclusions

In conclusion, marked differences were observed in genomic alterations, somatic interactions, pivotal signaling pathways, functional biological information, and accumulations of serum heavy metals between advanced NSCLC patients with and without BoM, and certain heavy metals (e.g., Cu, Sr) might have potentials to be risk factors for the occurrence of BoM.

## Supplementary Material

Supplementary figures.

## Figures and Tables

**Table 1:** Characteristics of patients with advanced NSCLC according to BoM.

Clinical characteristics	No. of patients	BoM		P-value
		Yes	No	
Total sample	109	67	42	
Age, years				
≤50	6	2	4	0.305
>50	103	65	38	
Gender				
Male	58	35	23	0.797
Female	51	32	19	
Histology				
LUAD	105	65	40	1.000
LUSC	4	2	2	
*EGFR* status				
Positive	64	40	24	0.792
Negative	45	27	18	
*TP53* status				
Positive	72	46	26	0.469
Negative	37	21	16	
History of smoking				
Yes	36	21	15	0.637
No	73	46	27	
History of drinking				
Yes	26	16	10	0.993
No	83	51	32	
History of pulmonary infection				
Yes	58	37	21	0.595
No	51	30	21	
TTF-1				
Positive	93	57	36	0.986
Negative	13	8	5	
Unknown	3	2	1	
Napsin A				
Positive	81	51	30	0.625
Negative	21	12	9	
Unknown	7	4	3	
CA-125 at baseline				
Normal	47	30	17	0.827
Elevated	39	24	15	
Unknown	23	13	10	
CA-153 at baseline				
Normal	58	35	23	0.371
Elevated	27	19	8	
Unknown	24	13	11	
CA-199 at baseline				
Normal	66	37	29	0.018*
Elevated	24	20	4	
Unknown	19	10	9	
CEA at baseline				
Normal	29	13	16	0.035*
Elevated	68	46	22	
Unknown	12	8	4	

NSCLC, non-small cell lung cancer; TTF-1, thyroid transcription factor 1; CA-125, cancer antigen 125; CA-153, cancer antigen 153; CA-199, cancer antigen 199; CEA, carcinoembryonic antigen. * p < 0.05

## References

[1] Sung H, Ferlay J, Siegel RL, Laversanne M, Soerjomataram I, Jemal A (2021). Global Cancer Statistics 2020: GLOBOCAN Estimates of Incidence and Mortality Worldwide for 36 Cancers in 185 Countries. CA Cancer J Clin.

[2] Herbst RS, Morgensztern D, Boshoff C (2018). The biology and management of non-small cell lung cancer. Nature.

[3] Santini D, Barni S, Intagliata S, Falcone A, Ferrau F, Galetta D (2015). Natural History of Non-Small-Cell Lung Cancer with Bone Metastases. Sci Rep.

[4] Kuchuk M, Kuchuk I, Sabri E, Hutton B, Clemons M, Wheatley-Price P (2015). The incidence and clinical impact of bone metastases in non-small cell lung cancer. Lung Cancer.

[5] Kuchuk M, Addison CL, Clemons M, Kuchuk I, Wheatley-Price P (2013). Incidence and consequences of bone metastases in lung cancer patients. J Bone Oncol.

[6] Hsiao KC, Chu PY, Chang GC, Liu KJ (2020). Elevated Expression of Lumican in Lung Cancer Cells Promotes Bone Metastasis through an Autocrine Regulatory Mechanism. Cancers (Basel).

[7] Ettinger DS, Aisner DL, Wood DE, Akerley W, Bauman J, Chang JY (2018). NCCN Guidelines Insights: Non-Small Cell Lung Cancer, Version 5.2018. J Natl Compr Canc Netw.

[8] Ettinger DS, Wood DE, Aggarwal C, Aisner DL, Akerley W, Bauman JR (2019). NCCN Guidelines Insights: Non-Small Cell Lung Cancer, Version 1.2020. J Natl Compr Canc Netw.

[9] Widding A, Stilbo I, Hansen SW, Hansen HH, Rossing N (1990). Scintigraphy with nanocolloid Tc 99m in patients with small cell lung cancer, with special reference to bone marrow and hepatic metastasis. Eur J Nucl Med.

[10] Song Q, Shang J, Zhang C, Zhang L, Wu X (2019). Impact of the homogeneous and heterogeneous risk factors on the incidence and survival outcome of bone metastasis in NSCLC patients. J Cancer Res Clin Oncol.

[11] da Silva GT, Bergmann A, Thuler LCS (2019). Incidence and Risk Factors for Bone Metastasis in Non-Small Cell Lung Cancer. Asian Pac J Cancer Prev.

[12] Li Y, Xu C, Yu Q (2022). Risk factor analysis of bone metastasis in patients with non-small cell lung cancer. Am J Transl Res.

[13] Deng K, Li S, Zhang J, Ye X, Yao K, Li Y (2022). Prognostic value of thoracic tumor staging and volume parameters in non-small cell lung cancer patients with synchronous solitary bone metastasis. J Thorac Dis.

[14] Zhang C, Mao M, Guo X, Cui P, Zhang L, Xu Y (2019). Nomogram based on homogeneous and heterogeneous associated factors for predicting bone metastases in patients with different histological types of lung cancer. BMC Cancer.

[15] Oliveira MB, Mello FC, Paschoal ME (2016). The relationship between lung cancer histology and the clinicopathological characteristics of bone metastases. Lung Cancer.

[16] Shan Q, Li Z, Lin J, Guo J, Han X, Song X (2020). Tumor Primary Location May Affect Metastasis Pattern for Patients with Stage IV NSCLC: A Population-Based Study. J Oncol.

[17] He JJ, Zhi K, Liu GF (2011). Predictive value of serum bone sialoprotein in patients with bone metastasis of non-small cell lung cancer. Onkologie.

[18] Zhang L, Hou X, Lu S, Rao H, Hou J, Luo R (2010). Predictive significance of bone sialoprotein and osteopontin for bone metastases in resected Chinese non-small-cell lung cancer patients: a large cohort retrospective study. Lung Cancer.

[19] Huff MO, Todd SL, Smith AL, Elpers JT, Smith AP, Murphy RD (2016). Arsenite and Cadmium Activate MAPK/ERK via Membrane Estrogen Receptors and G-Protein Coupled Estrogen Receptor Signaling in Human Lung Adenocarcinoma Cells. Toxicol Sci.

[20] Hartwig A, Asmuss M, Ehleben I, Herzer U, Kostelac D, Pelzer A (2002). Interference by toxic metal ions with DNA repair processes and cell cycle control: molecular mechanisms. Environ Health Perspect.

[21] Kim HS, Kim YJ, Seo YR (2015). An Overview of Carcinogenic Heavy Metal: Molecular Toxicity Mechanism and Prevention. J Cancer Prev.

[22] Caffo M, Caruso G, Fata GL, Barresi V, Visalli M, Venza M (2014). Heavy metals and epigenetic alterations in brain tumors. Curr Genomics.

[23] Diez M, Cerdan FJ, Arroyo M, Balibrea JL (1989). Use of the copper/zinc ratio in the diagnosis of lung cancer. Cancer.

[24] Jin Y, Zhang C, Xu H, Xue S, Wang Y, Hou Y (2011). Combined effects of serum trace metals and polymorphisms of CYP1A1 or GSTM1 on non-small cell lung cancer: a hospital based case-control study in China. Cancer Epidemiol.

[25] Oyama T, Matsuno K, Kawamoto T, Mitsudomi T, Shirakusa T, Kodama Y (1994). Efficiency of serum copper/zinc ratio for differential diagnosis of patients with and without lung cancer. Biol Trace Elem Res.

[26] Fracchia A, Ubbiali A, El Bitar O, Pacetti M, Sommariva E, Arreghini M (1999). A comparative study on ferritin concentration in serum and bilateral bronchoalveolar lavage fluid of patients with peripheral lung cancer versus control subjects. Oncology.

[27] Shi HB, Li XD, Jiang JT, Zhao WQ, Ji M, Wu CP (2014). Serum ferritin is elevated in advanced non-small cell lung cancer patients and is associated with efficacy of platinum-based chemotherapy. J Cancer Res Ther.

[28] Liu Y, Zhang N, Wang Y, Xu M, Liu N, Pang X (2012). Zinc finger E-box binding homeobox 1 promotes invasion and bone metastasis of small cell lung cancer in vitro and in vivo. Cancer Sci.

[29] Rinaldi S, Santoni M, Leoni G, Fiordoliva I, Marcantognini G, Meletani T (2019). The prognostic and predictive role of hyponatremia in patients with advanced non-small cell lung cancer (NSCLC) with bone metastases. Support Care Cancer.

[30] Shen H, Li Y, Liao Y, Zhang T, Liu Q, Du J (2012). Lower blood calcium associates with unfavorable prognosis and predicts for bone metastasis in NSCLC. PLoS One.

[31] Travis WD, Brambilla E, Nicholson AG, Yatabe Y, Austin JHM, Beasley MB (2015). The 2015 World Health Organization Classification of Lung Tumors: Impact of Genetic, Clinical and Radiologic Advances Since the 2004 Classification. J Thorac Oncol.

[32] Gandevia B, Tovell A (1964). Declaration of Helsinki. Med J Aust.

[33] Li H, Durbin R (2010). Fast and accurate long-read alignment with Burrows-Wheeler transform. Bioinformatics.

[34] Lai Z, Markovets A, Ahdesmaki M, Chapman B, Hofmann O, McEwen R (2016). VarDict: a novel and versatile variant caller for next-generation sequencing in cancer research. Nucleic Acids Res.

[35] Wang K, Li M, Hakonarson H (2010). ANNOVAR: functional annotation of genetic variants from high-throughput sequencing data. Nucleic Acids Res.

[36] Adzhubei IA, Schmidt S, Peshkin L, Ramensky VE, Gerasimova A, Bork P (2010). A method and server for predicting damaging missense mutations. Nat Methods.

[37] Schwarz JM, Rodelsperger C, Schuelke M, Seelow D (2010). MutationTaster evaluates disease-causing potential of sequence alterations. Nat Methods.

[38] Yu G, Wang LG, Han Y, He QY (2012). clusterProfiler: an R package for comparing biological themes among gene clusters. OMICS.

[39] Pao W, Wang TY, Riely GJ, Miller VA, Pan Q, Ladanyi M (2005). KRAS mutations and primary resistance of lung adenocarcinomas to gefitinib or erlotinib. PLoS Med.

[40] Mu D, Tang H, Teng G, Li X, Zhang Y, Gao G (2023). Differences of genomic alterations and heavy metals in non-small cell lung cancer with different histological subtypes. J Cancer Res Clin Oncol.

[41] Knapp BJ, Devarakonda S, Govindan R (2022). Bone metastases in non-small cell lung cancer: a narrative review. J Thorac Dis.

[42] Hendriks LE, Smit EF, Vosse BA, Mellema WW, Heideman DA, Bootsma GP (2014). EGFR mutated non-small cell lung cancer patients: more prone to development of bone and brain metastases?. Lung Cancer.

[43] Doebele RC, Lu X, Sumey C, Maxson DA, Weickhardt AJ, Oton AB (2012). Oncogene status predicts patterns of metastatic spread in treatment-naive nonsmall cell lung cancer. Cancer.

[44] Patil T, Mushtaq R, Marsh S, Azelby C, Pujara M, Davies KD (2020). Clinicopathologic Characteristics, Treatment Outcomes, and Acquired Resistance Patterns of Atypical EGFR Mutations and HER2 Alterations in Stage IV Non-Small-Cell Lung Cancer. Clin Lung Cancer.

[45] Fujimoto D, Ueda H, Shimizu R, Kato R, Otoshi T, Kawamura T (2014). Features and prognostic impact of distant metastasis in patients with stage IV lung adenocarcinoma harboring EGFR mutations: importance of bone metastasis. Clin Exp Metastasis.

[46] Navid S, Fan C, P OF-V, Generali D, Li Y (2020). The Fibroblast Growth Factor Receptors in Breast Cancer: from Oncogenesis to Better Treatments. Int J Mol Sci.

[47] Gartside MG, Chen H, Ibrahimi OA, Byron SA, Curtis AV, Wellens CL (2009). Loss-of-function fibroblast growth factor receptor-2 mutations in melanoma. Mol Cancer Res.

[48] Ruotsalainen T, Joensuu H, Mattson K, Salven P (2002). High pretreatment serum concentration of basic fibroblast growth factor is a predictor of poor prognosis in small cell lung cancer. Cancer Epidemiol Biomarkers Prev.

[49] Jing P, Zhao N, Xie N, Ye M, Zhang Y, Zhang Z (2018). miR-24-3p/FGFR3 Signaling as a Novel Axis Is Involved in Epithelial-Mesenchymal Transition and Regulates Lung Adenocarcinoma Progression. J Immunol Res.

[50] Kim HS, Kim JH, Jang HJ, Han B, Zang DY (2019). Pathological and Prognostic Impacts of FGFR2 Overexpression in Gastric Cancer: A Meta-Analysis. J Cancer.

[51] Ricol D, Cappellen D, El Marjou A, Gil-Diez-de-Medina S, Girault JM, Yoshida T (1999). Tumour suppressive properties of fibroblast growth factor receptor 2-IIIb in human bladder cancer. Oncogene.

[52] Helsten T, Elkin S, Arthur E, Tomson BN, Carter J, Kurzrock R (2016). The FGFR Landscape in Cancer: Analysis of 4,853 Tumors by Next-Generation Sequencing. Clin Cancer Res.

[53] Jaakkola S, Salmikangas P, Nylund S, Partanen J, Armstrong E, Pyrhonen S (1993). Amplification of fgfr4 gene in human breast and gynecological cancers. Int J Cancer.

[54] Peifer M, Fernandez-Cuesta L, Sos ML, George J, Seidel D, Kasper LH (2012). Integrative genome analyses identify key somatic driver mutations of small-cell lung cancer. Nat Genet.

[55] Latko M, Czyrek A, Porebska N, Kucinska M, Otlewski J, Zakrzewska M (2019). Cross-Talk between Fibroblast Growth Factor Receptors and Other Cell Surface Proteins. Cells.

[56] Mori S, Tran V, Nishikawa K, Kaneda T, Hamada Y, Kawaguchi N (2013). A dominant-negative FGF1 mutant (the R50E mutant) suppresses tumorigenesis and angiogenesis. PLoS One.

[57] Zou L, Cao S, Kang N, Huebert RC, Shah VH (2012). Fibronectin induces endothelial cell migration through beta1 integrin and Src-dependent phosphorylation of fibroblast growth factor receptor-1 at tyrosines 653/654 and 766. J Biol Chem.

[58] Hulit J, Suyama K, Chung S, Keren R, Agiostratidou G, Shan W (2007). N-cadherin signaling potentiates mammary tumor metastasis via enhanced extracellular signal-regulated kinase activation. Cancer Res.

[59] Nguyen T, Mege RM (2016). N-Cadherin and Fibroblast Growth Factor Receptors crosstalk in the control of developmental and cancer cell migrations. Eur J Cell Biol.

[60] Tsang M, Friesel R, Kudoh T, Dawid IB (2002). Identification of Sef, a novel modulator of FGF signalling. Nat Cell Biol.

[61] He Q, Gong Y, Gower L, Yang X, Friesel RE (2016). Sef Regulates Epithelial-Mesenchymal Transition in Breast Cancer Cells. J Cell Biochem.

[62] Lonic A, Barry EF, Quach C, Kobe B, Saunders N, Guthridge MA (2008). Fibroblast growth factor receptor 2 phosphorylation on serine 779 couples to 14-3-3 and regulates cell survival and proliferation. Mol Cell Biol.

[63] Gao G, Tian Z, Zhu HY, Ouyang XY (2018). miRNA-133b targets FGFR1 and presents multiple tumor suppressor activities in osteosarcoma. Cancer Cell Int.

[64] Bockorny B, Rusan M, Chen W, Liao RG, Li Y, Piccioni F (2018). RAS-MAPK Reactivation Facilitates Acquired Resistance in FGFR1-Amplified Lung Cancer and Underlies a Rationale for Upfront FGFR-MEK Blockade. Mol Cancer Ther.

[65] Huang GK, Huang CC, Kang CH, Cheng YT, Tsai PC, Kao YH (2023). Genetic Interference of FGFR3 Impedes Invasion of Upper Tract Urothelial Carcinoma Cells by Alleviating RAS/MAPK Signal Activity. Int J Mol Sci.

[66] Kondo T, Zheng L, Liu W, Kurebayashi J, Asa SL, Ezzat S (2007). Epigenetically controlled fibroblast growth factor receptor 2 signaling imposes on the RAS/BRAF/mitogen-activated protein kinase pathway to modulate thyroid cancer progression. Cancer Res.

[67] Das TK, Cagan RL (2017). KIF5B-RET Oncoprotein Signals through a Multi-kinase Signaling Hub. Cell Rep.

[68] Shanbhag V, Jasmer-McDonald K, Zhu S, Martin AL, Gudekar N, Khan A (2019). ATP7A delivers copper to the lysyl oxidase family of enzymes and promotes tumorigenesis and metastasis. Proc Natl Acad Sci U S A.

[69] Tsang T, Posimo JM, Gudiel AA, Cicchini M, Feldser DM, Brady DC (2020). Copper is an essential regulator of the autophagic kinases ULK1/2 to drive lung adenocarcinoma. Nat Cell Biol.

[70] Cobanoglu U, Demir H, Sayir F, Duran M, Mergan D (2010). Some mineral, trace element and heavy metal concentrations in lung cancer. Asian Pac J Cancer Prev.

[71] Eguchi R, Fujimori Y, Takeda H, Tabata C, Ohta T, Kuribayashi K (2011). Arsenic trioxide induces apoptosis through JNK and ERK in human mesothelioma cells. J Cell Physiol.

[72] Potin S, Bertoglio J, Breard J (2007). Involvement of a Rho-ROCK-JNK pathway in arsenic trioxide-induced apoptosis in chronic myelogenous leukemia cells. FEBS Lett.

[73] Yajima I, Uemura N, Nizam S, Khalequzzaman M, Thang ND, Kumasaka MY (2012). Barium inhibits arsenic-mediated apoptotic cell death in human squamous cell carcinoma cells. Arch Toxicol.

[74] Wingren G, Axelson O (1993). Epidemiologic studies of occupational cancer as related to complex mixtures of trace elements in the art glass industry. Scand J Work Environ Health.

[75] Adzhubei I, Jordan DM, Sunyaev SR (2013). Predicting functional effect of human missense mutations using PolyPhen-2. Curr Protoc Hum Genet.

[76] Genomes Project C, Auton A, Brooks LD, Durbin RM, Garrison EP, Kang HM (2015). A global reference for human genetic variation. Nature.

[77] Kircher M, Witten DM, Jain P, O'Roak BJ, Cooper GM, Shendure J (2014). A general framework for estimating the relative pathogenicity of human genetic variants. Nat Genet.

[78] Ng PC, Henikoff S (2003). SIFT: Predicting amino acid changes that affect protein function. Nucleic Acids Res.

[79] McNulty SN, Parikh BA, Duncavage EJ, Heusel JW, Pfeifer JD (2019). Optimization of Population Frequency Cutoffs for Filtering Common Germline Polymorphisms from Tumor-Only Next-Generation Sequencing Data. J Mol Diagn.

[80] Sukhai MA, Misyura M, Thomas M, Garg S, Zhang T, Stickle N (2019). Somatic Tumor Variant Filtration Strategies to Optimize Tumor-Only Molecular Profiling Using Targeted Next-Generation Sequencing Panels. J Mol Diagn.

[81] Hiltemann S, Jenster G, Trapman J, van der Spek P, Stubbs A (2015). Discriminating somatic and germline mutations in tumor DNA samples without matching normals. Genome Res.

[82] Teer JK, Zhang Y, Chen L, Welsh EA, Cress WD, Eschrich SA (2017). Evaluating somatic tumor mutation detection without matched normal samples. Hum Genomics.

[83] Huang X, Shi X, Huang D, Li B, Lin N, Pan W (2021). Mutational characteristics of bone metastasis of lung cancer. Ann Palliat Med.

