# Supplementary material for: Differences in Genomic Alterations and Accumulations of Heavy Metals Between Advanced Non-small Cell Lung Cancer Patients with and without Bone Metastasis
Source: J Cancer. 2024 Jun 3;15(13):4205–18. doi: 10.7150/jca.95191 (PMC11212080; doi:10.7150/jca.95191)
Supplement: Supplementary file 1 — Supplementary figures. [file jcav15p4205s1.pdf]

Differences in Genomic Alterations and Accumulations of Heavy Metals Between Advanced Non-small Cell Lung Cancer Patients with and without Bone Metastasis

Zhong-Qiang Yao, MD[1,†]; Hui-Hui Jiang, PhD[2,†] ✉; Fei-Fei Wang, BS[1]; Zhi-Gang Fan, PhD[1]; Yi-Ge Zhang, MD[1]; Shang-Dong Mou, MD[1]; Xia Cao, BS[1]; Cheng-Tian Li, BS[1]; Li-Sha Jiang, MD[2]; Li Song, MD[2]; Shu-Shen Ji, MD[2]; Qing-Juan Chen, PhD[1] ✉

1. Medical oncology, 3201 Hospital of Xi´an Jiaotong University Health Science Center,

   Hanzhong 723000, China.

2. Zhangjiang Center for Translational Medicine, Shanghai Biotecan Pharmaceuticals Co., Ltd., Shanghai 200135, China.

[†]These authors have contributed equally to this work.

✉ Corresponding authors: Qingjuan Chen (phone: 86-0916-2383463; e-mail: chenqingjuan2023@163.com) and Huihui Jiang (phone: 86-021-50277725; e-mail: jianghh@biotecan.com).

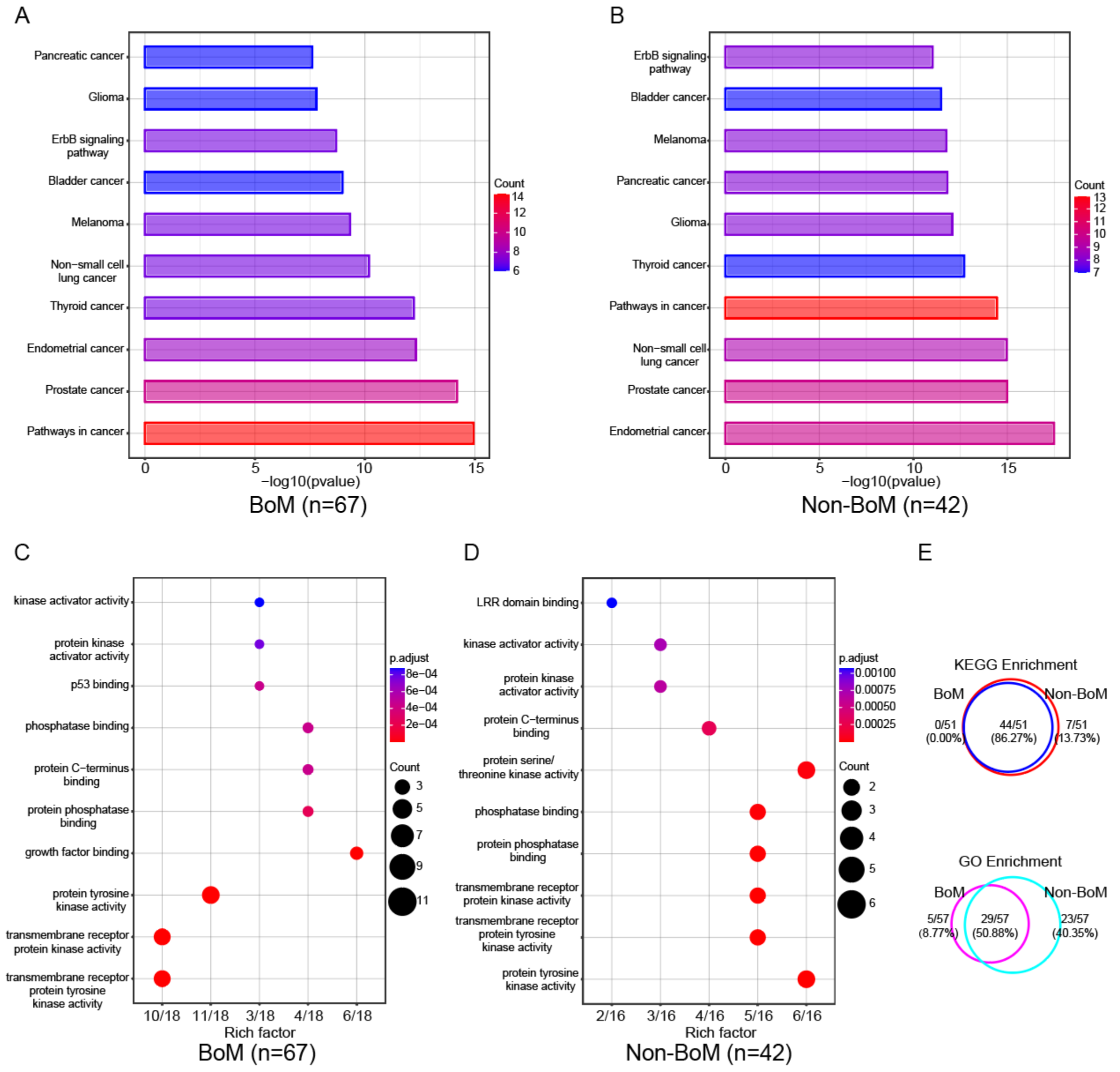

Supplementary figure 1. Signaling pathways and functional terms by KEGG (A, B) and GO (C, D) enrichment analysis in the BoM group (n=67) (A, C) and the non-BoM group (n=42) (B, D). Venn diagrams of signaling pathways (E, up) and functional terms (E, down) in advanced NSCLC patients with and without BoM.

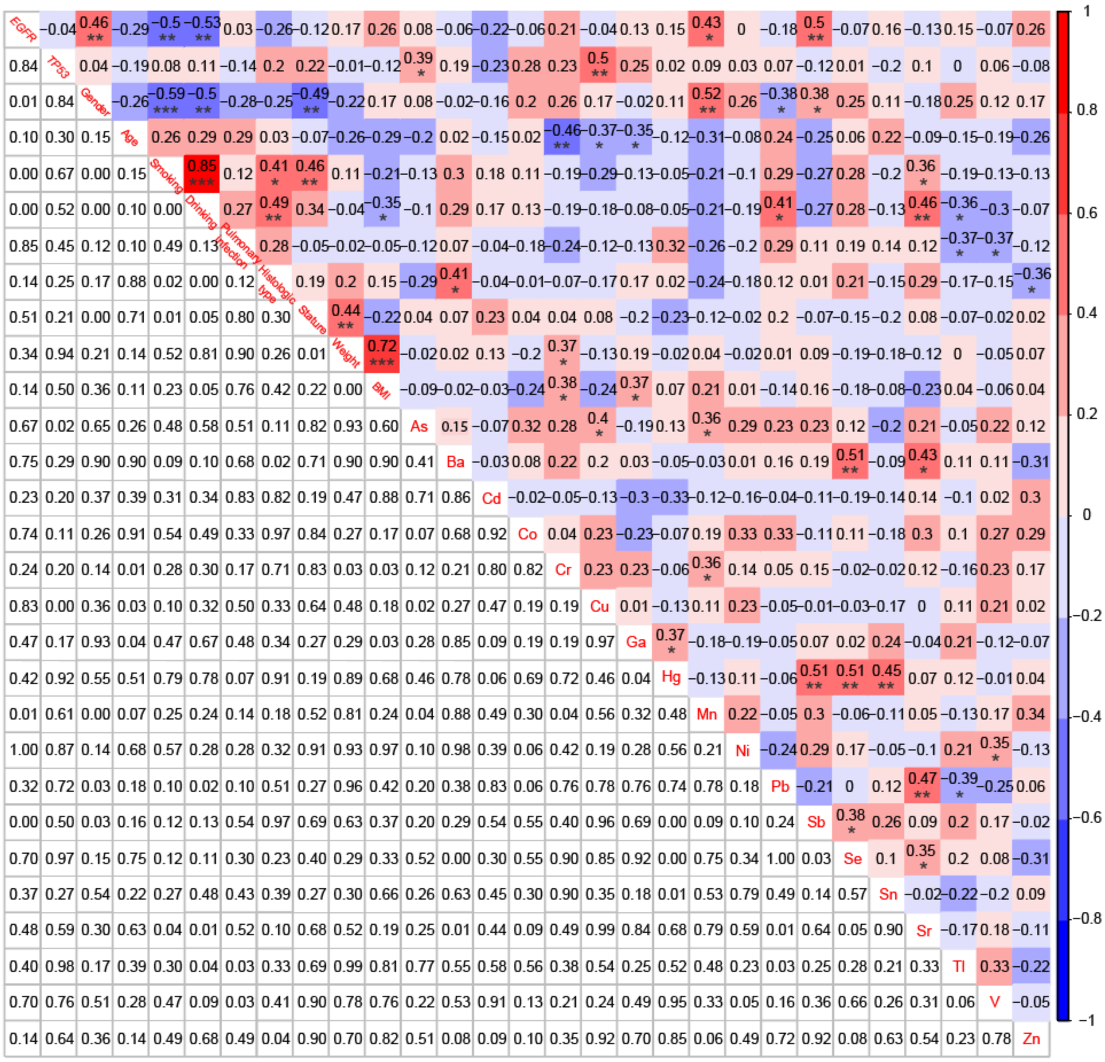

Supplementary figure 2. Correlations among *EGFR* mutations, *TP53* mutations, 9 demographic and clinical characteristics, and 18 heavy metals in advanced NSCLC patients without BoM (n=33). *p < 0.05, **p < 0.01, and ***p < 0.001.
